# Obesity Induced by High-Fat Diet Affects Epididymal Sperm Motility by Regulating Macrophage Polarization

**DOI:** 10.3390/biomedicines14081690

**Published:** 2026-07-28

**Authors:** Yafei Kang, Peiling Li, Xue Zhang, Xinyi Dong, Suying Yuan, Xu Wen, Zhixuan Yang, Xiaoxue Yang, Xiaoqing Zhang, Hu Zhao, Yonghong Tian, Donghui Huang

**Affiliations:** 1 Institute of Reproduction Health Research, Tongji Medical College, Huazhong University of Science and Technology, Wuhan 430030, China; kangyafeitt@163.com (Y.K.);; 2 The Second Clinical School, Tongji Hospital, Tongji Medical College, Huazhong University of Science and Technology, Wuhan 430030, China; 3 Department of Human Anatomy, Tongji Medical College, Huazhong University of Science and Technology, Wuhan 430030, China; 4 National Demonstration Center for Experimental Basic Medical Education, Huazhong University of Science and Technology, Wuhan 430030, China; 5 Department of Reproductive Endocrinology, Women’s Hospital, School of Medicine, Zhejiang University, Hangzhou 310000, China; 6 Shenzhen Huazhong University of Science and Technology Research Institute, Huazhong University of Science and Technology, Shenzhen 518109, China

**Keywords:** high-fat diet, sperm motility, polarization of macrophage, pyruvate carboxylase

## Abstract

**Objectives:** This study aimed to investigate the mechanisms underlying impaired sperm motility in high-fat diet (HFD)-induced obesity. **Methods**: Six-week-old male C57BL/6J mice were used to establish an HFD-induced obesity model. Sperm motility and male reproductive function were then evaluated. Macrophage polarization was evaluated by flow cytometry, and key differentially expressed genes were identified by RNA-seq analysis of epididymal adipose tissue, the epididymis, and testes. Finally, pyruvate carboxylase (PC) and the PC inhibitor erianin (Eri) were used to examine the role of adipocyte-derived PC in regulating macrophage polarization. **Results**: HFD-induced obese mice exhibited abnormal testicular and epididymal morphology, with reduced sperm motility and viability. Flow cytometry revealed that macrophages were polarized toward the M1 phenotype in epididymal adipose tissue and the epididymis in the HFD group. RNA-seq analysis demonstrated that *PC* was the most significantly upregulated gene in epididymal adipose tissue. Furthermore, *PC* expression increased during adipocyte maturation in vitro. Mature adipocytes promoted macrophage polarization toward the M1 phenotype, while Eri attenuated this effect. In addition, supernatants from LPS-induced M1 macrophages contained elevated levels of inflammatory cytokines and significantly reduced sperm motility and viability. **Conclusions**: HFD-induced obesity impaired sperm quality and male reproductive function. The underlying mechanism may involve increased PC expression, which drives epididymal macrophages toward M1 polarization and triggers local chronic inflammation, ultimately compromising sperm motility and viability.

## 1. Introduction

Obesity has become a serious public health concern in China and poses a major threat to global health. Globally, approximately 17% of men were obese in 2020, with 22 million classified as severely obese. By 2030, this proportion is expected to reach 20%, encompassing 34 million severely obese individuals [1]. Komninos et al. [2] reported that obesity interferes with spermatogenesis, thereby impairing male fertility and reproductive capacity. Clinical epidemiological studies [3] and animal models [4] have consistently confirmed that obesity negatively affects sperm quality. Furthermore, body mass index (BMI) is significantly and negatively associated with semen quality and male fertility [5,6,7,8].

Previous studies have reported that obesity represents a systemic, chronic, low-grade inflammatory state induced by various inflammatory mediators [9]. Macrophages constitute the most abundant immune cells in adipose tissue, comprising approximately 50% of the total cell population. Hypertrophic adipocytes recruit and activate macrophages, thereby exacerbating local inflammation [10]. In the obese state, lipid accumulation induces adipocyte apoptosis, leading to the release of elevated levels of free fatty acids and endotoxins. These factors subsequently promote macrophage polarization toward the proinflammatory M1 phenotype, increase proinflammatory cytokine release, and reduce anti-inflammatory cytokine production [11]. Inflammatory cytokines secreted by M1-polarized adipose tissue macrophages (ATMs) further promote macrophage recruitment, maintain the M1 phenotype, and establish a vicious inflammatory cycle [12]. Consequently, obesity often results in increased M1 macrophage polarization, creating a chronic inflammatory microenvironment within adipose tissue [13].

In the male reproductive system, macrophages play a crucial immunoregulatory role. Within the epididymis, macrophages exhibit clear regional specificity and are predominantly located in the caput epididymidis, suggesting their involvement in establishing the local immune microenvironment required for sperm maturation [14]. Specifically, M1 macrophages exhibit a proinflammatory phenotype and secrete cytokines such as interleukin-6 (IL-6), tumor necrosis factor-α (TNF-α), and interleukin-1β (IL-1β), thereby contributing to chronic inflammation. In contrast, M2 macrophages exhibit anti-inflammatory and tissue-repair properties. Elevated levels of proinflammatory cytokines, such as IL-6 and TNF-α, in semen have been strongly associated with reduced sperm quality and male infertility [15,16]. Paira et al. [17] reported that exposure of sperm to varying concentrations of IL-1β significantly decreased sperm viability and motility. Pascarelli et al. [18] demonstrated that TNF-α treatment adversely affected sperm motility, viability, mitochondrial function, and DNA integrity, whereas inhibition of TNF-α counteracted these harmful effects in vitro. Furthermore, Wang et al. [19] found that LPS-induced epididymitis impaired male fertility primarily through TNF-α production, an effect that was abolished in *Tnf*−/− mice.

In this study, an HFD-induced obese male mouse model was established to evaluate the effects of obesity on male reproductive function, macrophage polarization, and sperm quality and to explore the underlying mechanisms. The findings of this study may provide theoretical evidence and identify potential targets for interventions aimed at mitigating obesity-associated reproductive impairment.

## 2. Materials and Methods

### 2.1. Animal Model Establishment

Six-week-old male C57BL/6J mice were purchased from Shulaibao Biotechnology (Wuhan, China) and housed in the animal facility of the Institute of Reproductive Health. The room was maintained at 18–22 °C and 40–70% relative humidity under a 12-h light/dark cycle, with food and water available ad libitum. All mice were acclimated for at least 3–5 days before the experiments.

Mice were fed an HFD continuously for 14 weeks to establish a model of obesity and metabolic disorder. All experimental procedures involving animals were approved by the Experimental Animal Ethics Committee of Huazhong University of Science and Technology (Approval No. S1188) and conducted in strict accordance with animal welfare guidelines. After modeling was completed, fasting blood glucose and lipid profiles were measured the next day. Mice were euthanized by cervical dislocation, and epididymal adipose tissue, liver, spleen, testes, and epididymides were collected and weighed. The epididymal fat index, calculated as epididymal adipose tissue weight divided by body weight, was determined. For sperm quality analysis, the epididymis was incised using ophthalmic scissors, and sperm were gently expressed. Morphological examination was performed using hematoxylin and eosin (H&E) staining. Epididymal adipose tissue, epididymis, and testicular tissues were submitted to Wuhan Bioeasy Technology Co., Ltd. (Wuhan, China) for RNA-seq transcriptome sequencing.

### 2.2. Flow Cytometry Analysis of Macrophages and Subtype Proportions

Epididymal adipose tissue, epididymis, and testicular tissues were dissected, minced, and enzymatically digested with collagenase. Digestion was terminated by adding fetal bovine serum (FBS). Cell pellets were resuspended in PBS, counted, and filtered through a 300-mesh nylon filter into flow cytometry tubes. Blank control and single-stain control tubes each contained 5 × 10^5^ cells, whereas sample tubes contained 1 × 10^6^ cells. Cells were centrifuged at 800× *g* for 5 min at 4 °C, and the supernatants were discarded. Pellets were resuspended in 100 μL PBS. Fluorophore-conjugated antibodies against F4/80-FITC, CD11c-PE-Cy7, and CD206-APC were added to the corresponding tubes and mixed gently. After incubation for 30 min at room temperature in the dark, 1 mL PBS was added, and cells were centrifuged again at 800× *g* for 5 min at 4 °C. The supernatants were removed, and the pellets were resuspended in 200 μL PBS for flow cytometric analysis.

### 2.3. Adipocyte Differentiation and Co-Culture with Macrophages

Mouse 3T3-L1 preadipocytes were cultured in vitro. After reaching contact inhibition, the cells were cultured for an additional 48 h, which was defined as day 0 (D0). The culture medium was then replaced with differentiation medium I, consisting of complete high-glucose DMEM supplemented with 5 μg/mL insulin (Yeasen), 1 μmol/L dexamethasone (BioLegend), and 0.5 mmol/L IBMX (MCE). The medium was changed every two days for 4 days until D4 and then replaced with differentiation medium II, consisting of complete high-glucose DMEM supplemented with 5 μg/mL insulin, for an additional 2 days until D6. Subsequently, cells were cultured for another 4 days until D10 in complete high-glucose DMEM supplemented with 1% penicillin–streptomycin and 10% FBS, at which time they exhibited a mature adipocyte phenotype. For Oil Red O staining, cells at D2, D4, D6, D8, and D10 were fixed in 4% paraformaldehyde for 30 min, rinsed, washed briefly with 60% isopropanol for 10 s, stained with freshly prepared Oil Red O working solution for 1 h at room temperature in the dark, washed again, and observed under a microscope.

For co-culture experiments, 3T3-L1 adipocytes (1 × 10^5^ cells/well) were seeded at the bottom of 24-well plates, and RAW264.7 cells (1 × 10^5^ cells/well) were seeded in cell culture inserts placed within the same plates. After co-culturing for 24 h, RAW264.7 macrophages were collected by centrifugation and analyzed (e.g., by flow cytometry).

### 2.4. RNA Extraction, Reverse Transcription, and Quantitative Real-Time PCR

Total RNA was extracted from epididymal adipose tissue, Caput epididymis tissue, and 3T3-L1 adipocytes using the TRIzol-chloroform method. Reverse transcription was conducted using a commercial kit (Yeasen) according to the manufacturer’s instructions, and the resulting cDNA was immediately used for quantitative real-time PCR (qPCR). qPCR was performed using Hieff™ qPCR SYBR Green Master Mix (High Rox). Each reaction had a total volume of 20 μL, consisting of 10 μL SYBR Green Master Mix, 0.4 μL forward primer (10 μM), 0.4 μL reverse primer (10 μM), 1 μL cDNA, and 8.2 μL RNase-free water. PCR reactions were conducted using the StepOne Plus™ real-time PCR system (LongGene) under the following conditions: initial denaturation at 95 °C (5 min), followed by 40 cycles of 95 °C (10 s), 60 °C (20 s), and 72 °C (20 s). All samples were run in triplicate. Relative mRNA expression levels of target genes were calculated using the 2^−ΔΔCt^ method and normalized to β-actin as an internal reference. Primer sequences used in this study are listed in Table 1.

### 2.5. Human Sperm Samples

Normal human semen samples were obtained from the Reproductive Medicine Center of Tongji Medical College, Huazhong University of Science and Technology, and processed according to the WHO Laboratory Manual for the Examination and Processing of Human Semen (2021) Semen was ejaculated into sterile disposable containers and allowed to liquefy at 37 °C for at least 30 min. After liquefaction, the samples were centrifuged at 800× *g* for 5 min to remove seminal plasma. The sperm concentration was then adjusted to 3 × 10^7^ sperm/mL in Ham’s F-10 medium for each experiment. The separated seminal plasma was collected for subsequent co-incubation experiments.

### 2.6. Statistical Analysis

Data are expressed as mean ± standard error of the mean (SEM). Statistical analyses were performed using GraphPad Prism 9.0 software. Comparisons between two independent groups were conducted using the unpaired *t*-test. Comparisons among multiple groups were conducted using one-way analysis of variance (ANOVA). Statistical significance was defined as *p* < 0.05, with significance levels indicated as follows: * *p* < 0.05, ** *p* < 0.01, *** *p* < 0.001, **** *p* < 0.0001, and ns for non-significant differences.

## 3. Results

### 3.1. HFD Induces Systemic Metabolic Disorders in Male Mice

**Figure 1:**
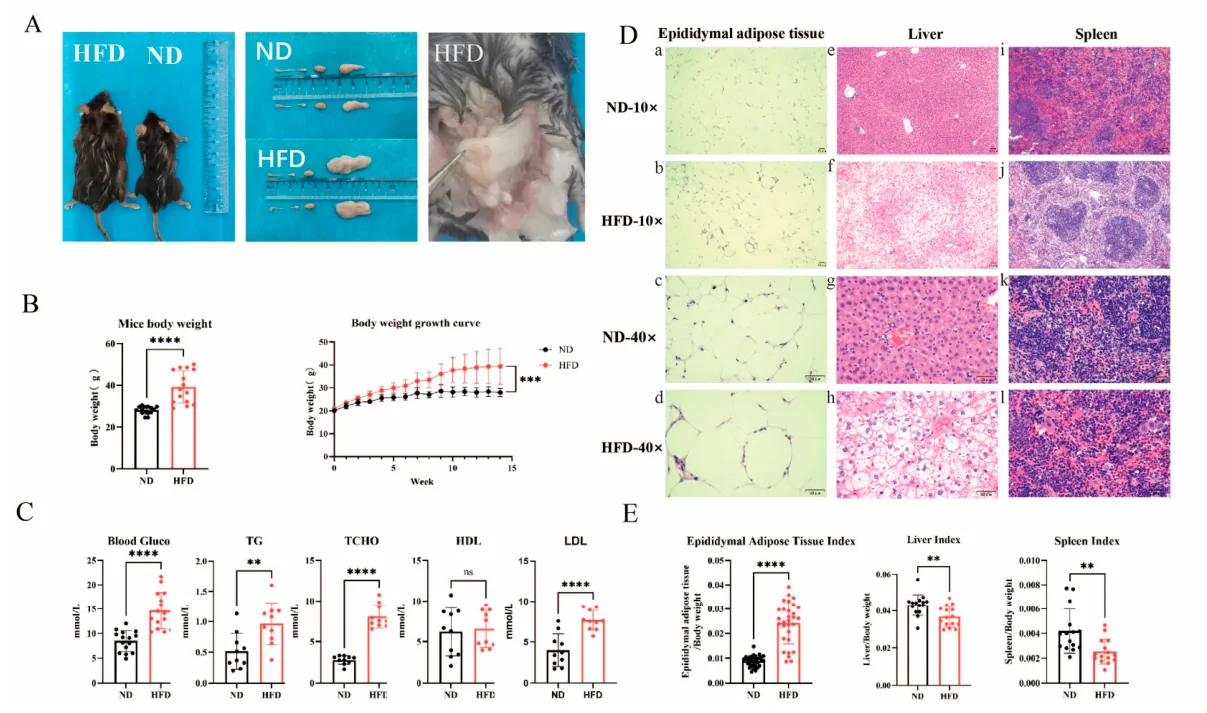
HFD induced systemic metabolic disorders in male mice. (**A**) Anatomical comparisons between the HFD and ND groups: overall appearance; sizes of the epididymis, testis, and epididymal adipose tissue; anatomical relationship between the epididymis and epididymal adipose tissue. (**B**) Body weight and growth curves after 14 weeks of dietary intervention. (**C**) Fasting blood glucose and lipid profiles (TG, TC, LDL, HDL). (**D**) H&E staining of epididymal adipose tissue (**a**–**d**), liver (**e**–**h**), and spleen (**i**–**l**) from ND and HFD-fed mice (scale bars: 10× and 40× magnification). (**E**) Epididymal fat index (epididymal adipose tissue weight/body weight), liver index (liver weight/body weight), and spleen index (spleen weight/body weight) (** *p* < 0.01, *** *p* < 0.001, **** *p* < 0.0001; ns, not significant).

Compared with mice in the normal diet (ND) group, HFD-fed mice exhibited marked obesity. Epididymal adipose tissue was markedly enlarged and closely surrounded the caput epididymidis (Figure 1A,B). Levels of fasting blood glucose, triglycerides (TGs), total cholesterol (TC), and low-density lipoprotein (LDL) were significantly increased (*p* < 0.05, Figure 1C). Epididymal fat indices were significantly higher, whereas liver and spleen indices were significantly decreased (Figure 1E). Histological examination revealed enlarged adipocytes with irregular morphology and increased intercellular spaces in the epididymal adipose tissue of the HFD group. Hepatocytes showed vacuoles of variable size with ballooning degeneration, and the splenic sinusoids were dilated, with a looser cellular arrangement.

### 3.2. HFD-Induced Obesity Reduces Sperm Motility and Impairs Reproductive Function

**Figure 2:**
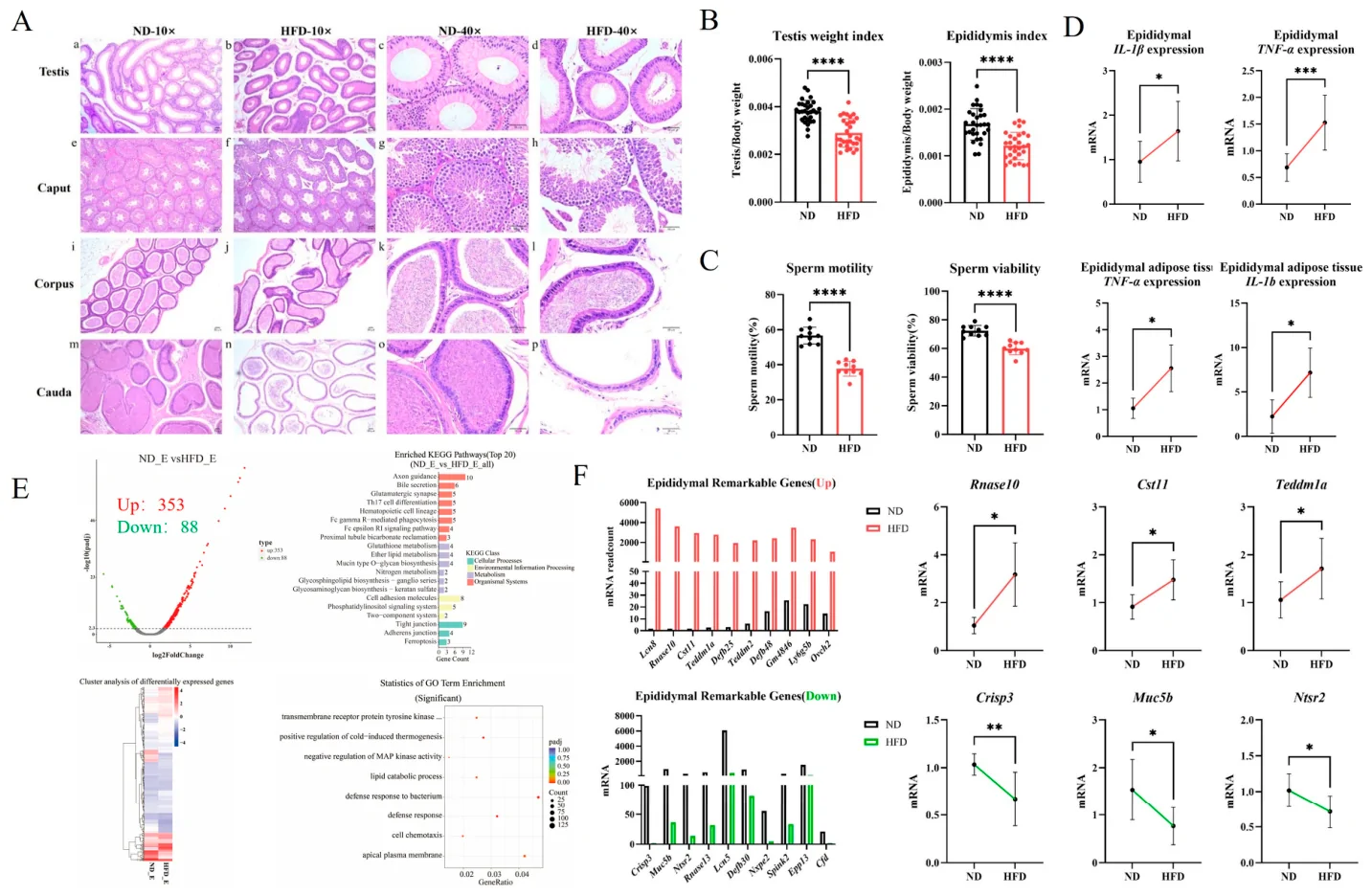
HFD impairs testicular and epididymal structure and reduces sperm quality. (**A**) H&E staining of testis (**a**–**d**), epididymal head (**e**–**h**), body (**i**–**l**), and tail (**m**–**p**) from ND and HFD-fed mice (scale bars: 10× and 40× magnification). (**B**) Testis weight, epididymal weight, testis index, and epididymal index (n = 15). (**C**) Sperm motility and viability (n = 10). (**D**) Expression of inflammatory genes in epididymal adipose and epididymal tissues from HFD mice. (**E**) Volcano plot, heatmap, and analysis of differentially expressed genes in the epididymis of HFD-fed mice. (**F**) RT-PCR validation of selected differentially expressed genes (n = 8) (* *p* < 0.05, ** *p* < 0.01, *** *p* < 0.001, **** *p* < 0.0001).

Compared with ND mice, HFD-fed mice showed no significant differences in testicular or epididymal weight. However, the testis and epididymis indices were significantly lower, and sperm motility and viability were markedly decreased (Figure 2B,C). H&E staining revealed fewer spermatogenic cells in testicular tissue, widened epididymal duct lumens, and an apparent reduction in the number of spermatozoa within the cauda epididymal lumens (Figure 2A). RNA-seq analysis identified 353 upregulated and 88 downregulated genes in the epididymis of HFD-fed mice relative to ND mice (Figure 2E). KEGG analysis revealed that the upregulated genes were enriched in axon guidance, tight junction, and leukocyte transendothelial migration pathways. Conversely, the downregulated genes were enriched in the PPAR signaling and NOD-like receptor signaling pathways (Figure 2B). Gene expression analysis of inflammatory factors demonstrated that HFD significantly increased the expression of *IL-1β* and *TNF-α* in epididymal adipose tissue and epididymal tissue (Figure 2D). GO analysis indicated that the upregulated genes were involved in responses to cytokines and the regulation of cold-induced thermogenesis, whereas the downregulated genes were primarily associated with defense responses to bacteria and immune responses (Figure 2E). RT-PCR validation of three significantly upregulated and three significantly downregulated genes showed results consistent with the sequencing data (Figure 2F).

### 3.3. Effect of HFD on Macrophage Polarization in Male Reproductive Tissues

**Figure 3:**
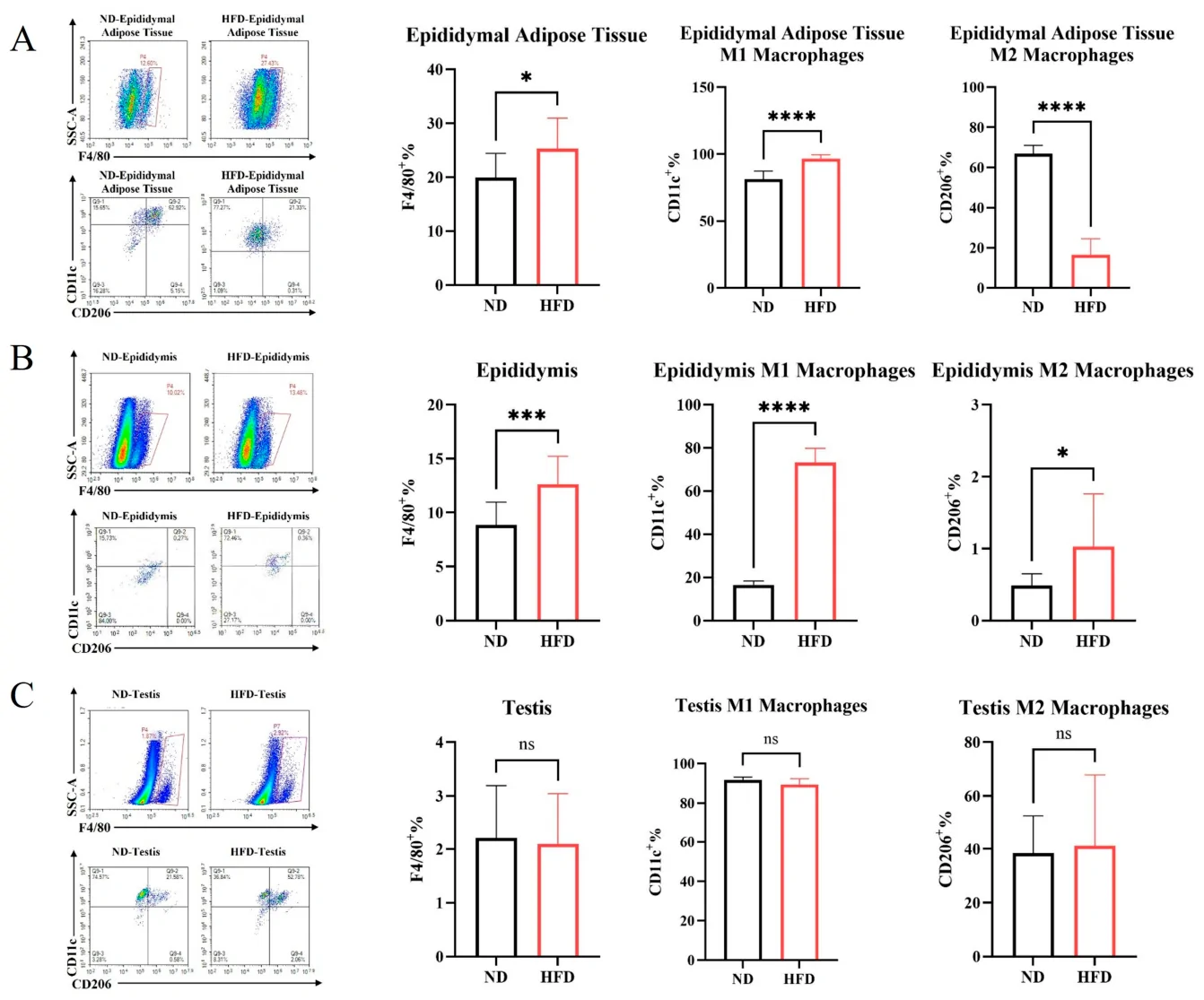
Effects of HFD on macrophages and polarization in male reproductive tissues. (**A**) Total macrophages and polarization proportions in epididymal adipose tissue of HFD vs. ND mice (n = 10). (**B**) Total macrophages and polarization proportions in the epididymis (n = 10). (**C**) Total macrophages and polarization proportions in the testis (n = 10) (* *p* < 0.05, *** *p* < 0.001, **** *p* < 0.0001; ns, not significant).

Flow cytometry analysis indicated that the proportion of total macrophages was significantly increased in the epididymal adipose tissue of HFD-fed mice. The proportion of M1 macrophages was significantly increased, whereas that of M2 macrophages was significantly decreased, resulting in a markedly elevated M1/M2 ratio (Figure 3A). In the epididymis, the proportion of total macrophages was also significantly increased. Both M1 and M2 populations increased; however, the increase in M1 macrophages was more pronounced, resulting in a significantly elevated M1/M2 ratio (Figure 3B). In contrast, no significant changes were observed in the proportion of total macrophages, in the proportions of M1 and M2 macrophages, or in the M1/M2 ratio in the testis (Figure 3C).

### 3.4. PC Expression Is Most Markedly Upregulated in Adipocytes of HFD-Fed Male Mice

**Figure 4:**
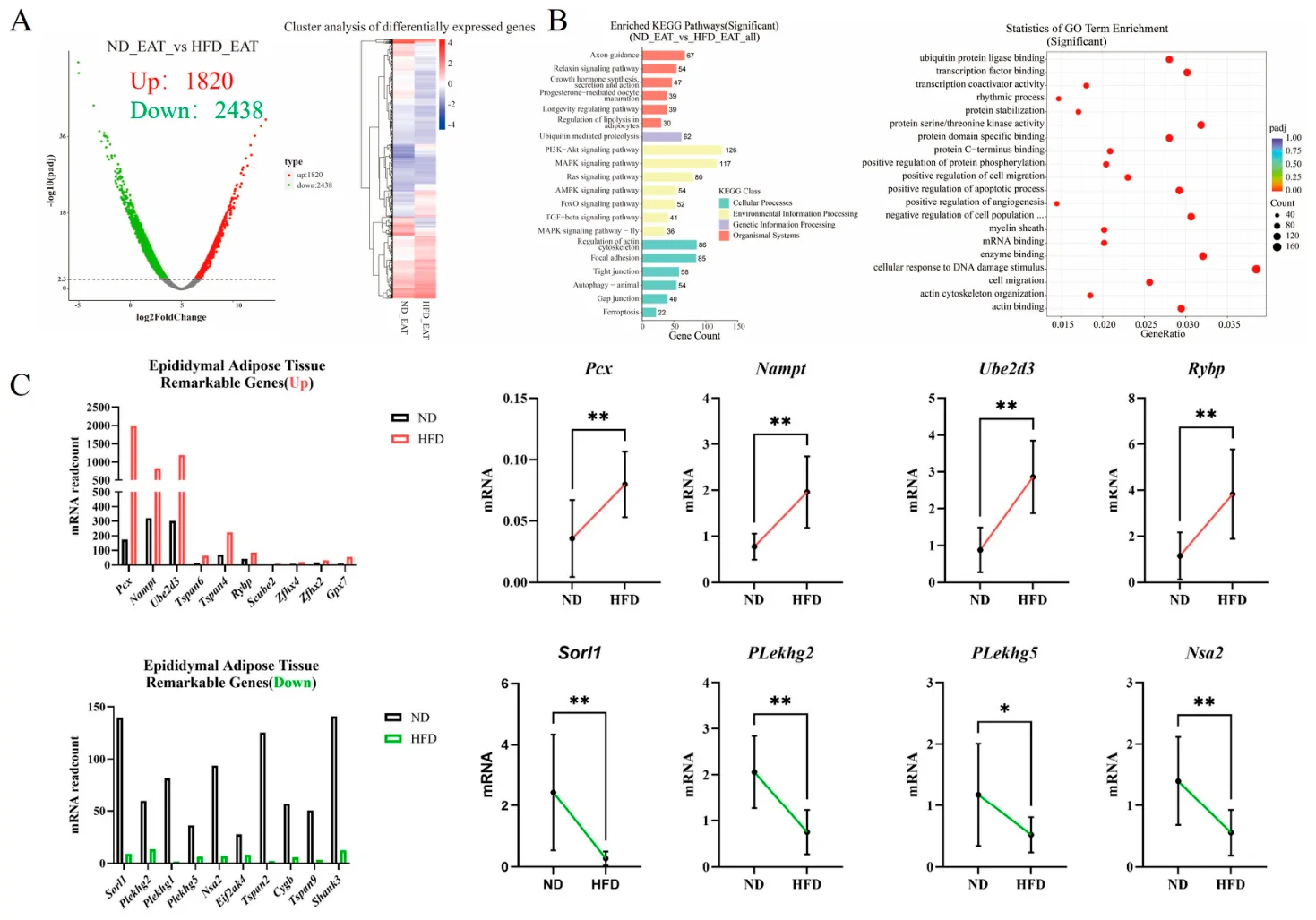
RNA-seq analysis and validation of differentially expressed genes in epididymal adipose tissue. (**A**) Volcano plot and heatmap (red: upregulated genes; blue: downregulated genes). (**B**) Pathway analysis of differentially expressed genes in epididymal adipose tissue from HFD-fed mice. (**C**) RT-qPCR validation of selected upregulated and downregulated genes (n = 8) (* *p* < 0.05, ** *p* < 0.01).

To identify key factors potentially driving macrophage polarization after adipose-tissue dysregulation, RNA-seq analysis was performed using epididymal adipose tissue. The analysis identified 1820 upregulated and 2438 downregulated genes in HFD-fed mice relative to ND mice (Figure 4A). Among these genes, PC showed the greatest increase in expression. KEGG enrichment analysis indicated that the upregulated genes were associated with pathways such as ubiquitin-mediated proteolysis, PI3K-Akt signaling, FOXO signaling, focal adhesion, and protein processing in the endoplasmic reticulum. Conversely, the downregulated genes were related to MAPK signaling, purine metabolism, regulation of the actin cytoskeleton, lysosome, PI3K-Akt signaling, and adipocytokine signaling pathways (Figure 4B). RT-qPCR validation of four selected differentially expressed genes yielded results consistent with the RNA-seq data (Figure 4C).

### 3.5. Effect of Adipocyte PC on Macrophage Polarization

**Figure 5:**
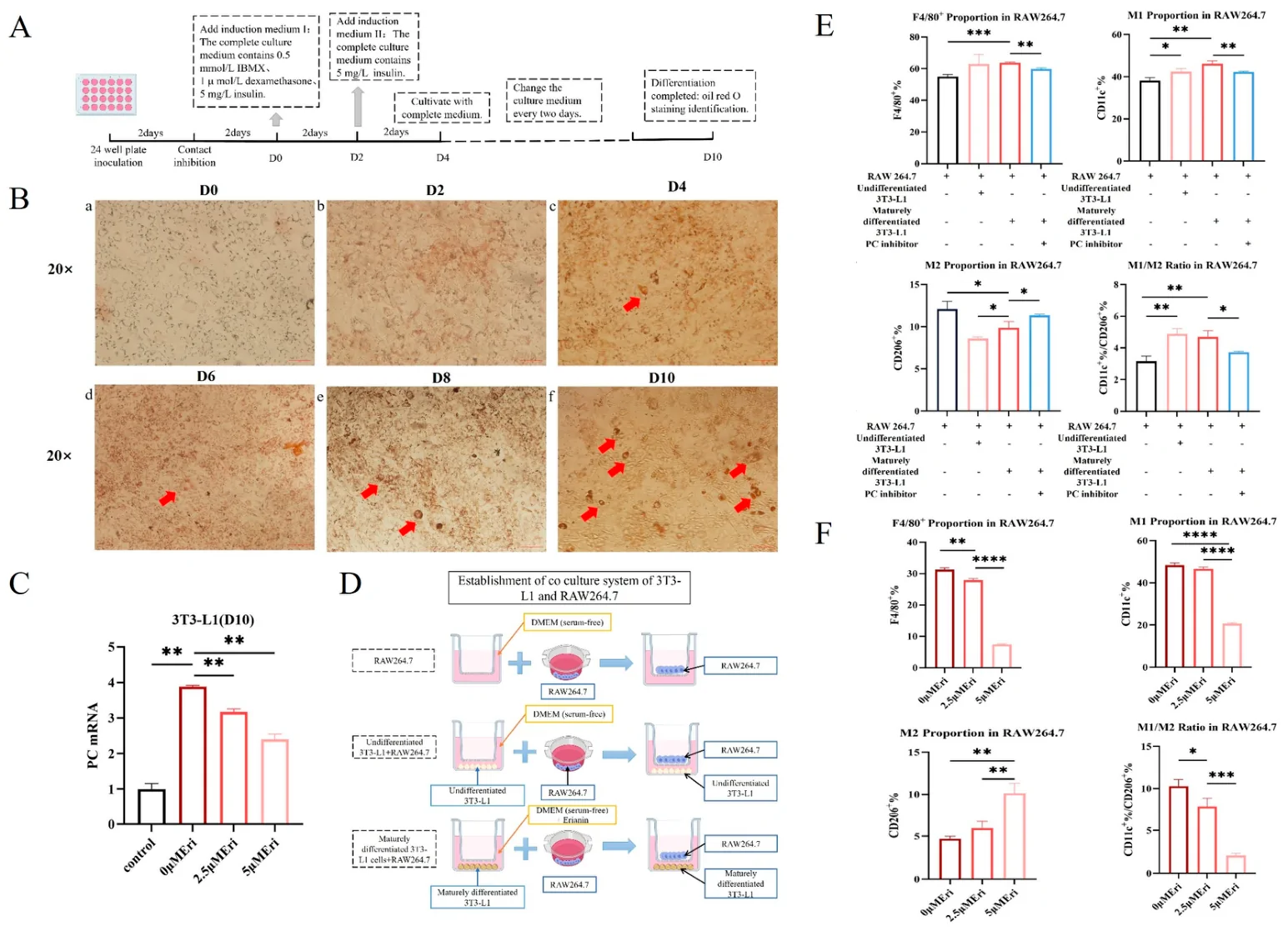
Regulatory effects of adipocyte-derived PC on macrophage polarization. (**A**) Differentiation protocol of 3T3-L1 adipocytes. (**B**) Oil Red O staining of 3T3-L1 cells at different stages of differentiation (red arrows indicate lipid droplets). (**C**) PC expression in 3T3-L1 cells treated with different concentrations of Eri (n = 3). (**D**) Schematic illustration of the co-culture system. (**E**) Effects of adipocyte-derived PC on macrophage number and polarization (n = 3). (**F**) Effects of different Eri concentrations (0 μM, 2.5 μM, and 5 μM) on macrophage number and polarization in the co-culture system (n = 3) (* *p* < 0.05, ** *p* < 0.01, *** *p* < 0.001, **** *p* < 0.0001).

To investigate the role of adipocyte-derived PC in macrophage polarization, 3T3-L1 preadipocytes were first induced to differentiate into mature adipocytes in vitro (Figure 5A). Before differentiation, lipid droplets were not evident in the cells. Lipid droplets began to appear on day 4, and by day 10, Oil Red O staining revealed abundant lipid-droplet accumulation, indicating successful adipocyte differentiation (Figure 5B). RT-qPCR analysis showed that *PC* mRNA expression was significantly increased after adipocyte maturation. In contrast, treatment with the PC inhibitor Eri markedly suppressed *PC* expression in a dose-dependent manner (Figure 5C). Subsequently, a co-culture system consisting of adipocytes (3T3-L1) and macrophages (RAW264.7) was established (Figure 5D). After 24 h of co-culture with mature adipocytes, the number of macrophages was significantly higher than that in the negative control group. This change was accompanied by an increased proportion of M1 macrophages, a decreased proportion of M2 macrophages, and a significantly elevated M1/M2 ratio. Notably, Eri treatment markedly attenuated adipocyte-induced M1 macrophage polarization (Figure 5E). Moreover, the inhibitory effect became more pronounced with increasing Eri concentrations (Figure 5F).

### 3.6. Effect of Macrophage Polarization on Sperm Quality

**Figure 6:**
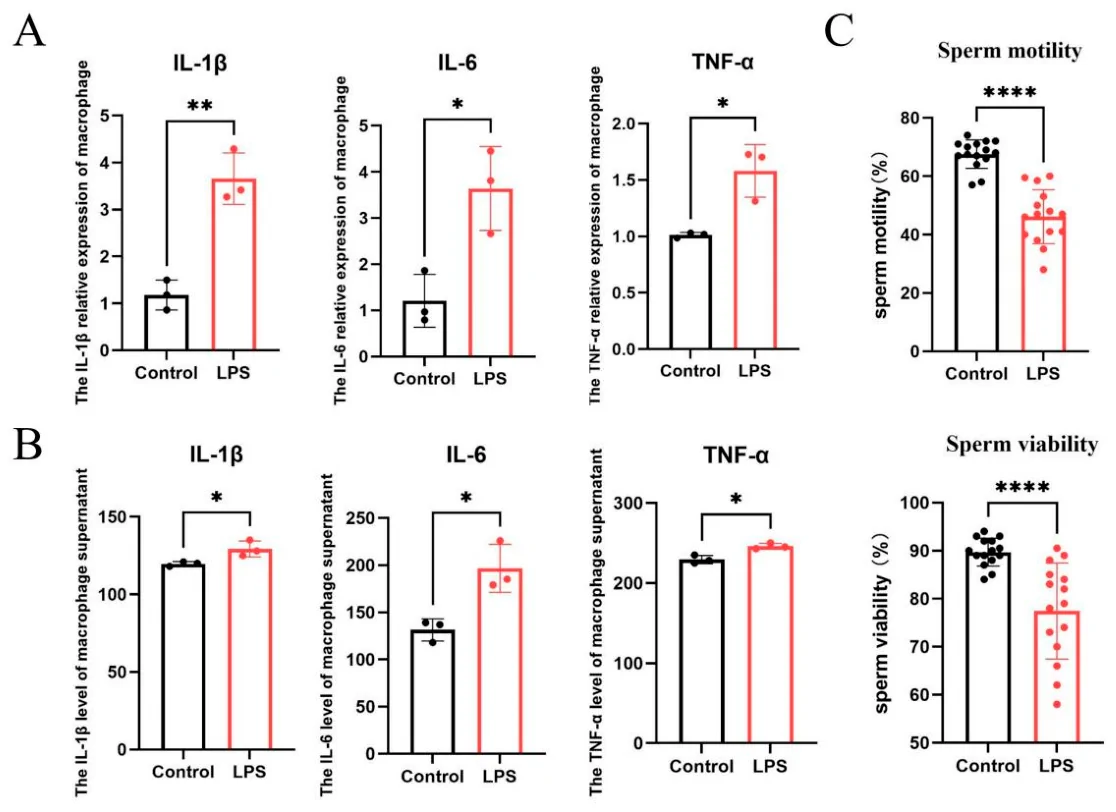
Effects of macrophage polarization on human sperm quality. (**A**,**B**) RAW264.7 macrophages treated with LPS (100 ng/mL, 24 h): A. Gene expression levels of inflammatory cytokines. B. Protein levels of inflammatory cytokines in culture supernatants. (**C**) Effects of conditioned supernatants from LPS-treated macrophages on normal human sperm motility and viability (* *p* < 0.05, ** *p* < 0.01. RAW: macrophages only; LPS: macrophages + LPS (100 ng/mL); ITA: macrophages + LPS (100 ng/mL) + ITA (10 μM, 24 h) (* *p* < 0.05, ** *p* < 0.01, **** *p* < 0.0001).

RAW264.7 macrophages were treated with LPS (100 ng/mL) for 24 h to induce M1 polarization. LPS treatment significantly increased the expression of proinflammatory cytokines, including *IL-6*, *TNF-α*, and *IL-1β* (Figure 6A). Consistently, the protein levels of these cytokines were also significantly elevated in the culture supernatants (Figure 6B). Furthermore, incubation of normal human sperm with conditioned supernatants from LPS-induced macrophages significantly reduced sperm motility and viability (Figure 6C).

## 4. Discussion

This study demonstrated that HFD-induced obesity impairs male reproductive function, particularly sperm quality. The underlying mechanism may involve upregulated expression of adipocyte-derived PC, which promotes M1 polarization of epididymal macrophages and subsequently induces local epididymal inflammation.

### 4.1. Effect of HFD-Induced Obesity on Male Reproductive Function

Although obesity has a complex etiology, HFD is considered one of the major contributors to obesity and metabolic syndrome in humans [20]. Epidemiological studies have demonstrated that excessive dietary fat intake is closely associated with reduced male fertility [21,22,23,24]. A 2013 study reported that men with high saturated fat intake exhibited a significantly lower sperm concentration and total sperm count, with a clear dose-dependent relationship [25]. Similarly, Çekici et al. [26] demonstrated that trans-fatty acid intake was negatively associated with sperm concentration and total sperm count and positively associated with asthenozoospermia. In addition, studies investigating fatty acid composition and sperm parameters found that oleic acid and monounsaturated fatty acids were negatively correlated with sperm concentration, progressive motility, normal morphology, sperm viability, and fertility index. However, these fatty acids were positively correlated with sperm necrosis. Sperm motility was also negatively associated with saturated fatty acid levels [27]. In this study, testicular and epididymal weights did not differ significantly between the HFD and ND groups. However, mice in the HFD group showed substantial decreases in caudal epididymal sperm count, sperm motility, and sperm viability. These results are in line with previous evidence showing that HFD feeding reduces sperm count, motility, and fertilizing capacity, even when testicular weight remains unchanged. Consistently, other studies have reported lower caudal epididymal sperm counts in mice fed an HFD, despite no apparent differences in testicular weight or histological morphology between groups [28]. Together, these findings indicate that sperm quantity and functional parameters are more sensitive indicators of obesity- and metabolism-related reproductive damage than gross reproductive organ weight. Thus, testicular or epididymal weight alone may not be sufficient to evaluate obesity-associated male reproductive dysfunction. In contrast, sperm quality-related parameters may provide a more accurate assessment of HFD-induced reproductive impairment.

Previous studies have shown that C57BL/6J mice fed an HFD are more susceptible to fat accumulation, weight gain, and glucose metabolism disorders than other mouse strains [29]. This model has several advantages, including a relatively short induction period and stable physiological characteristics, and is therefore widely used in obesity-related studies [30]. Deshpande et al. [31] reported that HFD-induced obese rats exhibited increased germ cell apoptosis in the testes, impaired fertility, and significantly reduced litter size. A meta-analysis conducted by Crean et al. [4] provided robust animal-based evidence supporting the detrimental effects of HFD on male fertility. Similarly, Luo et al. [32] demonstrated that HFD-induced metabolic disorders impaired spermatogenesis in rats. In this study, a mouse model of obesity was successfully established by feeding male C57BL/6J mice an HFD for 14 weeks. The results demonstrated significant metabolic abnormalities and substantial accumulation of epididymal adipose tissue. Further analyses revealed varying degrees of morphological damage in the testes and epididymides, accompanied by significantly reduced sperm motility and viability in the cauda epididymis. These findings indicate that HFD-induced obesity markedly impairs male reproductive function, which is consistent with previous studies.

Obesity is widely regarded as a state of chronic low-grade systemic inflammation, characterized by macrophage infiltration into adipose tissue and phenotypic remodeling [33,34]. Obesity impairs male fertility through multiple interrelated mechanisms, including reproductive endocrine dysfunction, increased local temperature, oxidative stress, chronic inflammation, and blood–testis barrier disruption. Previous studies have demonstrated that HFD-induced obese mice exhibit reduced serum testosterone, elevated estradiol levels, impaired blood–testis barrier integrity, and decreased sperm quality [35]. Therefore, reproductive hormone levels were not reassessed in the present study. In addition, obesity-associated fat accumulation and oxidative stress may further impair spermatogenesis and sperm function [36]. Although these mechanisms collectively contribute to obesity-associated male reproductive dysfunction, our findings suggest that local inflammation in epididymal adipose tissue may play a key role.

### 4.2. HFD-Induced Obesity Induces M1 Polarization of Epididymal Macrophages and Local Epididymal Inflammation

Obesity is strongly associated with chronic inflammation in adipose tissue, which is mainly driven by prolonged nutrient overload and subsequent immune activation [37]. Zhao et al. [38] reported that obesity-induced chronic inflammation in the testicular microenvironment of male mice damaged the blood–testis barrier, impaired Sertoli cell function, and ultimately disrupted spermatogenesis and steroidogenesis. Similarly, Fan et al. [35] demonstrated that diet-induced obesity disrupted the blood–testis barrier, activated the NLRP3 inflammasome, and increased the levels of pro-inflammatory cytokines, including IL-6 and TNF-α, thereby reducing fertility. Zhang et al. [39] found that obesity upregulated macrophage- and NLRP3 inflammasome-related genes and proteins in the testes, leading to chronic low-grade inflammation. Additionally, Zhao et al. [38] observed significant increases in serum TNF-α and IL-10, dyslipidemia, decreased sperm parameters, and pathological testicular changes in adult male mice fed a high-fat and high-protein diet.

The epididymis plays a crucial role in sperm maturation. Ding et al. [40] reported that HFD-induced obesity primarily decreased sperm motility, suggesting that the epididymis may be particularly vulnerable to HFD-induced metabolic stress. Epididymal tissue appears more susceptible than testicular tissue to inflammation and immune responses during reproductive tract infections [41]. Qu et al. [42] also indicated that inflammatory lesions in the epididymis are usually more severe than those in the testis. Moreover, RNA-seq analysis in this study revealed 1820 upregulated and 2438 downregulated differentially expressed genes in epididymal adipose tissue, 353 upregulated and 88 downregulated genes in the epididymis, and only one downregulated gene in the testis. These findings suggest that HFD-induced obesity affects the epididymis more substantially than the testis. Sequencing further showed that genes upregulated in the epididymis of obese mice were enriched in axon guidance, tight junction, and leukocyte transendothelial migration pathways, whereas downregulated genes were primarily associated with defense responses to bacteria and other immune defense mechanisms. RT-qPCR validation indicated increased expression of proinflammatory cytokines in epididymal tissues. These results suggest that HFD-induced reductions in sperm motility are associated with activation of inflammatory pathways and disruption of immune defense mechanisms in the epididymis.

Macrophages constitute the primary immune cell population in the epididymis. Under physiological conditions, reproductive tract macrophages predominantly exhibit the immunosuppressive M2 phenotype [43]. During infection-induced inflammation, macrophages shift toward the M1 phenotype and secrete proinflammatory cytokines such as IL-6, TNF-α, and IL-1β [44]. Lumeng et al. [43] observed that HFD-induced obesity promotes a phenotypic shift of macrophages from M2 to M1 in adipose tissue. Proinflammatory cytokines secreted by M1 macrophages further drive macrophage polarization toward the M1 phenotype, creating a vicious inflammatory cycle [2]. In the present study, HFD significantly promoted M1 polarization of macrophages in epididymal adipose tissue and the epididymis, but not in the testis. This finding suggests that HFD-induced obesity contributes to local epididymal inflammation, with more pronounced proinflammatory macrophage polarization in the epididymis than in the testis. Additionally, elevated *IL-1β* and *TNF-α* gene expression levels in the caput epididymidis further indicate that HFD-induced M1 macrophage polarization is closely associated with chronic inflammation.

### 4.3. Relationship Between Adipocyte-Derived PC and Macrophage Polarization

PC is a key mitochondrial anaplerotic enzyme that catalyzes the carboxylation of pyruvate to oxaloacetate, thereby replenishing intermediates of the tricarboxylic acid (TCA) cycle [45]. Increased PC expression may therefore affect downstream metabolic fluxes, including oxaloacetate-derived aspartate metabolism and citrate-dependent lipid synthesis [46]. PC is also closely related to lipid metabolism. Previous studies have reported increased PC activity in metabolic tissues under HFD-induced metabolic stress [47,48,49]. For example, Kumashiro et al. demonstrated that hepatic PC activity was elevated in HFD-fed rats, whereas treatment with PC-specific antisense oligonucleotides reduced obesity, plasma lipid levels, and hepatic steatosis [49]. These findings support a role for PC in glucose and lipid metabolic homeostasis and suggest that PC may be a potential therapeutic target for obesity-related metabolic disorders.

In addition to its metabolic function, PC may also participate in immunometabolic regulation. Recent studies have shown that macrophage PC promotes metabolic reprogramming and amplifies inflammatory responses through activation of the HIF-1 signaling pathway [50]. Aspartate metabolism has also been reported to enhance IL-1β production in inflammatory macrophages by promoting HIF-1α activation and NLRP3 inflammasome signaling [51]. Other TCA cycle intermediates may similarly function as immunometabolic signals during macrophage activation. For example, succinate stabilizes HIF-1α and induces IL-1β expression [52]. Citrate-derived acetyl-CoA, produced through ATP-citrate lyase, supports fatty acid synthesis and protein acetylation, thereby regulating inflammatory gene expression and macrophage activation [53,54]. On the basis of these observations, we speculate that PC upregulation in epididymal adipose tissue may drive macrophage polarization toward the M1 phenotype by reshaping the local metabolic microenvironment. This mechanism may involve oxaloacetate/aspartate metabolism, citrate-derived acetyl-CoA production, and HIF-1α-mediated inflammatory signaling. However, the specific PC-dependent metabolites that directly promote macrophage polarization in HFD-induced epididymal adipose tissue remain unclear and should be defined in future studies using targeted metabolomics or isotope-tracing experiments.

Considering that RNA-seq analysis of whole epididymal adipose tissue cannot determine whether PC upregulation occurs specifically in adipocytes or also in infiltrating macrophages and other stromal cells, future studies will include immunohistochemistry (IHC) and immunofluorescence (IF) analyses to define the cellular localization of PC protein in epididymal adipose tissue.

Previous studies have reported high PC expression during adipocyte differentiation and have implicated PC in adipocyte lipid metabolism. In 3T3-L1 cells, both PC activity and protein abundance increase substantially during adipogenic differentiation. PC has also been linked to lipid metabolism in human white adipocytes. These observations support adipocytes as a potential major source of PC in adipose tissue [55]. In addition, PC is closely associated with lipid synthesis [56], and increased PC activity has been detected in metabolic tissues under HFD conditions [47,48,49]. Kumashiro et al. [49] further showed that PC-specific antisense oligonucleotides reduced obesity, plasma lipid levels, and hepatic steatosis in HFD-fed rats, suggesting that PC may be an important metabolic target in obesity-related disorders. Evidence from recent studies also suggests that PC may function beyond metabolic regulation and participate in immunometabolic control. Shu et al. [57] reported that PC regulates CD8+ T-cell responses and macrophage M1 polarization and reduces the immunosuppressive activity of tumor-associated macrophages through PD-L1 downregulation. In addition, the PC inhibitor Eri suppresses NLRP3 inflammasome activation both in vitro and in vivo and shows therapeutic effects in models of gouty arthritis and HFD-induced type 2 diabetes [58]. These findings suggest that PC may couple metabolic remodeling to inflammatory regulation. Accordingly, PC upregulation in epididymal adipose tissue may contribute to HFD-induced macrophage M1 polarization and local inflammation, thereby impairing sperm motility and viability.

In this study, RNA sequencing identified PC as the most significantly upregulated gene in the epididymal adipose tissue of HFD-fed mice. Among the four genes selected for validation, namely *PC*, *Nampt*, *Ube2d3*, and *Rybp*, *PC* showed the greatest upregulation. Cell-based experiments revealed that *PC* expression progressively increased during adipocyte differentiation and maturation. Furthermore, co-culture with mature adipocytes markedly promoted macrophage polarization toward the M1 phenotype, and this effect was significantly reversed by Eri treatment. Given that the epididymis in HFD-induced obese mice is closely surrounded by epididymal adipose tissue and is anatomically adjacent and vascularly connected to this tissue, changes in the metabolic and immune microenvironment of epididymal adipose tissue may influence the local epididymal microenvironment. These changes may promote M1 macrophage polarization and increase inflammatory cytokine levels.

### 4.4. M1 Polarization of Macrophages Impairs Sperm Motility

Loveland et al. [59] demonstrated that hyperglycemia-induced inflammation and sperm damage in the male reproductive system were associated with macrophage M1 polarization. Zhu et al. [60] found that shifting macrophages from the M1 phenotype to the M2 phenotype alleviated reproductive inflammation, increased serum and testicular testosterone levels, and enhanced sperm motility in diabetic male mice. Additionally, exposure of sperm to different concentrations of IL-1β reduced sperm viability and motility [17]. Wang et al. [19] reported that LPS-induced epididymitis impairs male fertility primarily through increased TNF-α production, and this effect was abolished in TNF-α knockout mice. In the present study, LPS treatment induced significant M1 polarization of RAW264.7 macrophages. This change was accompanied by increased levels of *IL-6*, *TNF-α*, and *IL-1β* in cell lysates and culture supernatants. Moreover, incubation of normal human sperm with macrophage-conditioned supernatants markedly reduced sperm motility and viability. These findings further support the conclusion that macrophage M1 polarization in the reproductive system may adversely affect sperm quality and male fertility.

In the present study, LPS stimulation drove RAW264.7 macrophages toward an M1 phenotype, as indicated by significantly increased mRNA expression of the pro-inflammatory cytokine genes *Il6*, *Tnf*, and *Il1b* [61]. Conditioned medium from these M1-polarized macrophages significantly reduced the motility and viability of normal human sperm. This result is consistent with previous evidence that inflammatory cytokines, including *IL-1β*, can directly impair human sperm motility and viability [17]. LPS-induced epididymitis has also been shown to compromise testicular and epididymal function through increased TNF-α production, with adverse effects on male fertility [19]. However, the LPS-induced M1 macrophage system is an acute inflammatory model and does not fully reproduce the chronic metabolic inflammatory microenvironment associated with HFD-induced obesity. Direct treatment of sperm with epididymal fluid from HFD-fed mice, or with conditioned medium prepared from HFD epididymal tissues, would provide a more physiologically relevant model. Because sample availability was limited, the present experiments were designed primarily as mechanistic validation to test whether soluble inflammatory mediators released by M1 macrophages can directly damage sperm function. Together with the in vivo results, these findings support the interpretation that HFD-induced obesity promotes M1 macrophage polarization in epididymal adipose tissue and the epididymis, thereby creating a local pro-inflammatory microenvironment that contributes to impaired sperm maturation and motility. Future studies will compare the cytokine profiles of conditioned medium from LPS-induced M1 macrophages, epididymal fluid, and conditioned medium from HFD epididymal tissues and will further assess their direct effects on sperm function.

## 5. Conclusions

**Figure 7:**
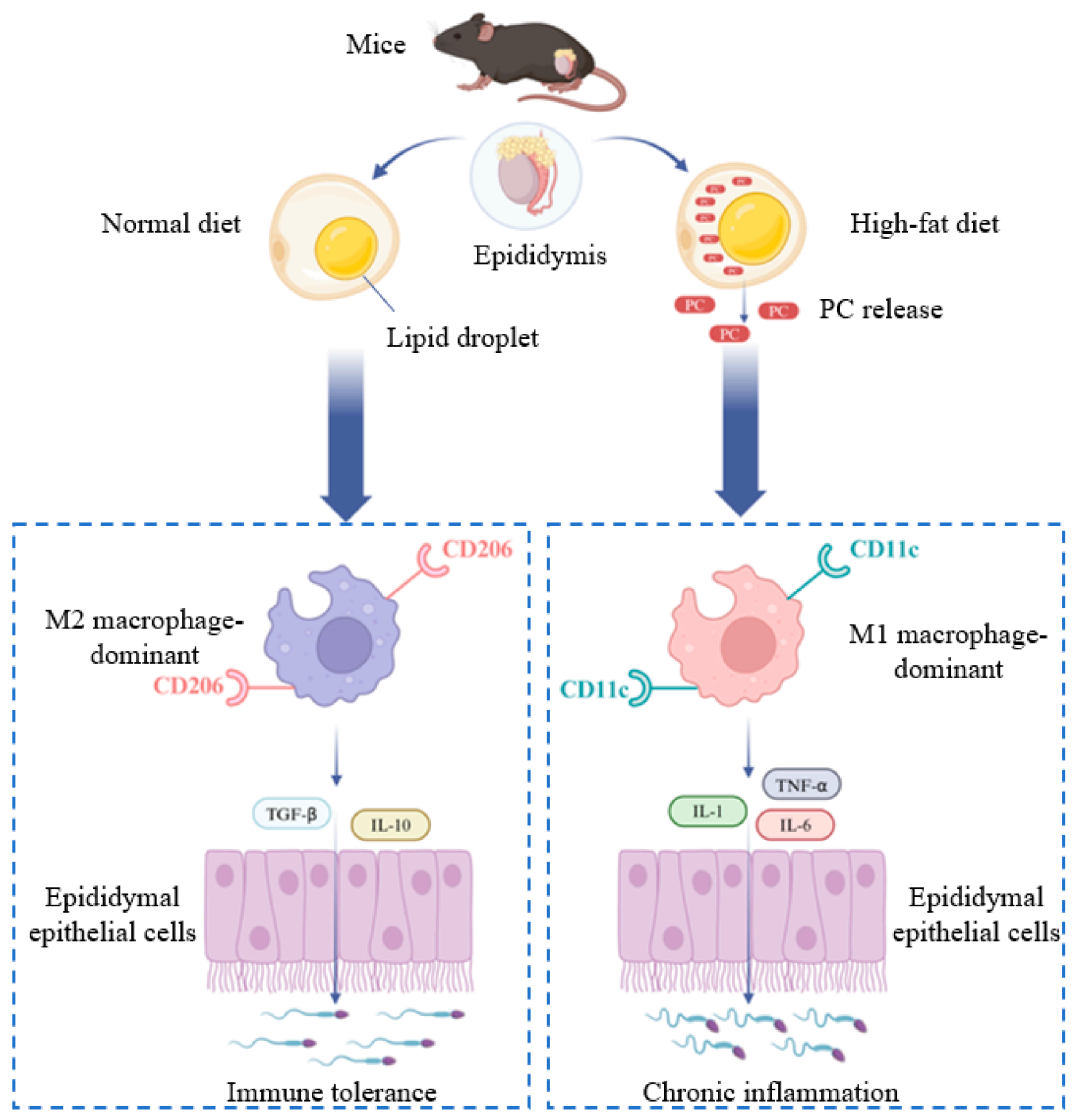
Proposed mechanism by which HFD-induced obesity affects epididymal macrophages through epididymal adipose tissue. HFD-induced obesity leads to a significant increase in PC expression in epididymal adipose tissue. This increase may promote macrophage polarization toward the M1 phenotype in the epididymis, induce local chronic inflammation, and ultimately impair sperm quality and male reproductive function.

In summary, HFD-induced impairment of male reproductive function may be attributed, at least in part, to chronic epididymal inflammation associated with M1 macrophage polarization. This process is closely associated with increased PC expression in epididymal adipose tissue. Adipocyte-derived PC may promote M1 macrophage polarization in the epididymis and trigger local chronic inflammation, thereby impairing sperm motility and male reproductive function (Figure 7).

This study investigated the effects of HFD on epididymal macrophages. The results demonstrated that HFD significantly promoted M1 macrophage polarization in both epididymal adipose tissue and the epididymis. This finding suggests that the decline in male fertility caused by HFD is closely associated with M1 macrophage polarization. However, macrophage polarization in testicular tissue was not significantly altered. This finding indicates that HFD-induced local inflammation predominantly occurs in the epididymis and epididymal adipose tissue, rather than in the testes. Given the close anatomical association between epididymal adipose tissue and the epididymis, metabolic alterations in epididymal adipose tissue may directly influence epididymal function. However, the exact mechanism by which PC derived from epididymal adipose tissue reaches the epididymis remains to be clarified and warrants further investigation. Overall, this study provides novel insights into the mechanisms linking adipose metabolism to male reproductive dysfunction. It also offers new perspectives for clinical research on male infertility and identifies potential therapeutic targets for obesity-associated reproductive impairment.

## Figures and Tables

**Table 1:** Primer sequences of mice.

Gene	Primer Sequences
Mouse-*PC*	F: CTGAAGTTCCAAACAGTTCGAGG
	R: CGCACGAAACACTCGGATG
Mouse-*Nampt*	F: CCTGGTATCCAATTACAGTGGC
	R: CCAAATGAGCAGATGCCCCTAT
Mouse-*Ube2d3*	F: CAGTAATGGCAGCATTTGTCTTG
	R: GGGTCGTCTGGGTTTGGAT
Mouse-*Rybp*	F: ACAACAAAGACCAGCGAAACAAACC
	R: GGATGTGGAGGAGGAGCGAGTC
Mouse-*Sorl1*	F: GAACACCTGTCTCCGAAACCAG
	R: CGGAACTGAGTGTCTGCATCAC
Mouse-*Plekhg2*	F: CCACCCACGACATTCCCAAGTTC
	R: AGGTCCTCCTCCTCCTCCTCTTC
Mouse-*Plekhg5*	F: TGGAGGAAGAAGAGGAAGGCTGTAC
	R: CTTGCTCGGGTCATCTGTGTGTC
Mouse-*Nsa2*	F: CCCAAAGTTCGTGCCCAAGGAG
	R: AAAGCCATCGCCAACAAAACAGAC
Mouse-*Rnase10*	F: GGACATAGGAAGAGCCAAGACTA
	R: GATGATCCTCCAGGACGGCAG
Mouse-*Cst11*	F: CACCTGCTTGAAGACAGAGACG
	R: CTTCAACCCAGGGAATGGCGTA
Mouse-*Teddm1a*	F: GTGTCTCTTCCTTTTGGCACCG
	R: AGCCTGGTCATCTTTGTCCTGC
Mouse-*Crisp3*	F: GTGGAGTTGCTGAATGCCCTG
	R: ATGGTGCTCTTGCTGTGTAAGCT

## Data Availability

The data supporting the findings of this study are available from the corresponding author upon reasonable request.
